# Editorial: Where to Raise Happy and Skilled Children: How Environment Shapes Human Development and Education

**DOI:** 10.3389/fpsyg.2020.594924

**Published:** 2020-11-30

**Authors:** Sabine Pirchio, Blanca Silvia Fraijo Sing, Ylenia Passiatore

**Affiliations:** ^1^Department of Dynamic and Clinical Psychology, Sapienza University of Rome, Rome, Italy; ^2^Department of Psychology and Communication Sciences, University of Sonora, Hermosillo, Mexico; ^3^Department of Education, Roma Tre University, Rome, Italy

**Keywords:** development, education, environment, child, adolescent, relationships, school

Child development consists of a series of changes that occur in the individual, driven by genetic, biological, social, cultural, and environmental resources and constraints (Bronfenbrenner, 1979; Hendry and Kloep, 2002). Child development has been described and explained through different perspectives, focusing on the changes in different components (e.g., cognition, emotion, relations, language, etc.) implying different developmental processes (e.g., continuous vs. discontinuous, quantitative vs. qualitative, maturational vs. social processes) and using different scientific methodologies (e.g., lab experiments, field observations, cross-sectional vs. longitudinal research designs).

This long research tradition has helped uncover the many ways in which a newborn grows into an adult, offering important insights into interventions, education, and social policies.

Following a basic assumption of environmental psychology, human beings, as all forms of living organisms, are shaped by the physical and social characteristics of their life's environments, impacting on the development of their skills, preferences, habits, and behaviors (Bonnes and Carrus, 2004; Mercado-Doménech et al., 2017). On the other hand, individuals and social groups leave a footprint on their habitats. The environment is, in some measure, an outcome of human actions (Gifford, 2011).

According to the ecological approach to human development (Bronfenbrenner, 1979), child development occurs in a series of hierarchically organized environmental systems, characterized by specific properties, components, and rules and linked by reciprocal and dynamic relations, and interactions. The social-relational aspects of development and the impact of specific activities for learning and acquisition represent the main research stream in this framework (e.g., Bruner, 1983; Tomasello et al., 1993; Pontecorvo and Pirchio, 2000).

Recently, however, psychologists have shifted their attention toward investigating the relationships among the physical properties of the environment and child development and behavior (Legendre, 2003; Evans, 2006; Carrus et al., 2015).

The articles included in this Research Topic contribute relevant knowledge about “where to raise happy and skilled children” from three different perspectives. In the first perspective, features of home and the school environment are juxtaposed with aspects of children's developmental processes, such as the development of gender stereotypes (Solbes-Canales et al.) and environmental attitudes and behaviors (Durón-Ramos et al.) and with developmental resources such as parental involvement in their children's education (Echeverría-Castro et al.). Rural and urban living environments are not only physically different, but foster and constrain the experiences adults and children could have in different ways, and may play a role in their happiness and well-being (Cerina and Fornara, 2011; Kabisch et al., 2017; Maricchiolo et al., 2020) through complex dynamics involving the environmental affordances and the people's behaviors and choices (Carrus et al., 2020). To be able to identify environmental features that play a role in children's development and behaviors certainly contributes to identifying and shaping positive educational environments (Tapia-Fonllem et al.).

A second perspective links six articles that shed light on the connectedness to nature and on the challenges and resources to improve the feeling of connection to nature in children in educational settings. Connectedness to nature is an important factor in environmental education as it is linked to pro-environmental behaviors (Liefländer et al., 2013). There is a robust tradition of environmental education trying to target connectedness to nature to have an impact on pro-environmental behavior (Passafaro et al., 2010; Otto and Pensini, 2017; Varela-Candamio et al., 2018), showing how complex it can be to create long-term effects in children's attitudes and behaviors toward nature and the environment. Although incomplete, preschool children already have a concept of nature (Fraijo-Sing et al.), and they attach their positive and negative emotional responses to nature (Olivos-Jara et al.); moreover, connectedness to nature is related to sustainable behaviors and happiness and related to the child's self-definition (Barrera-Hernández et al.). Given the relevance of the connection to nature for a child's well-being and for the environmental development, interventions aiming to increase connection to nature are important. The mini review by Barrable and Booth analyzes different types of interventions and identifies relevant variables to be considered for further research and for planning interventions, such as the age of participants and length of the contact with nature. Even if there could be several settings in which connection to nature could be increased, educational institutions, and schools in particular, seem to be the most important. The articles by Pérez-López et al. and van Dijk-Wesselius et al., address the issue of providing preschool and primary school teachers with the knowledge, attitudes, and confidence to implement educational activities involving nature. The research within this perspective highlights the need for more systematic studies on the effect of diverse types of experiences with nature on environmental attitudes and pro-environmental behaviors.

The studies in the third perspective address the cognitive side of the research on the outcomes of contact with nature. Natural environments have a restorative power. Being in contact with nature recharges an individual's cognitive and emotional resources (Kaplan and Kaplan, 1989; Hartig, 2004) in adults (Hartig et al., 2011; Carrus et al., 2017) and children (Hattie et al., 1997; Korpela, 2002; Carrus et al., 2015). Johnson et al. find effects of a nature intervention on children's endogenous attention, and Federico discusses the convergent influence of the natural environment and of social relationships on stress reduction and, consequently, on attentional processes.

Our Research Topic contributes to the study of environmental psychology by accumulating new knowledge about the ways in which physical qualities of educational environments influence children's cognitive functioning and social behavior, assessing instruments to measure relevant factors of child development in different living environments, and in discussing environmental interventions.

## Author Contributions

All authors listed have made a substantial, direct and intellectual contribution to the work, and approved it for publication.

## Conflict of Interest

The authors declare that the research was conducted in the absence of any commercial or financial relationships that could be construed as a potential conflict of interest.
